# The complete chloroplast genome of endangered Zhangjiajie sage *Salvia daiguii* Y. K. Wei & Y. B. Huang (Lamiaceae)

**DOI:** 10.1080/23802359.2020.1840934

**Published:** 2020-12-24

**Authors:** Xuan Zhou, Yan-Bo Huang, Zhi-Cheng Zhang, Xin-Yan Xu, Rui-Hong Wang, Ling Xu, Zhe-Chen Qi, Yu-Kun Wei

**Affiliations:** a Zhejiang Province Key Laboratory of Plant Secondary Metabolism and Regulation, College of Life Sciences and Medicine, Zhejiang Sci-Tech University, Hangzhou, PR China; b Shanghai Chenshan Plant Science Research Centre, Chinese Academy of Sciences, Shanghai Chenshan Botanical Garden, Shanghai, PR China

**Keywords:** *Salvia daiguii*, chloroplast genome, endangered species

## Abstract

The complete chloroplast genome of Zhangjiajie sage, *Salvia daiguii*, was assembled in this study. The genome is 151,434 bp in length and contained 134 encoded genes in total, including 88 protein-coding genes, eight ribosomal *RNA* genes, and 37 transfer *RNA* genes. The result of phylogenetic analysis based on 17 chloroplast genomes revealed that *S. daiguii* is clustered with *Salvia miltiorrhiza* in Lamiaceae.

Zhangjiajie sage, *Salvia daiguii* Y. K. Wei & Y. B. Huang, is a newly described species in *Salvia* subgenus *Glutinaria* (Wei et al. 2019). The species has a unique streamside habitat with limestone bedrock, and is only known from Wulingyuan area, Zhangjiajie National Forest Park, Hunan province, China. The discovered population comprises no more than four hundred mature individuals and the number show dramatic fluctuations from year to year. *S. daiguii* has been classified as ‘Critically Endangered’ (CR) according to IUCN guidelines and suggest that immediate measures should be taken to ensure the long-term survival of this species in its original habitat (Wei et al. 2019). It usually grows in a compact form with sub-leathery and glossy leaves, and inflorescence is prominent. Therefore, *S. daiguii* has potential value of ornamental horticulture. Additionally, recent study showed that root of *S. daiguii* has high composition of caffeic acid, salvianolic acid B, and rosmarinic acid (Wang 2020). However, no genetic resource has been reported for this CR species. In this study, the complete chloroplast genome of *S. daiguii* was assembled and annotated, which will provide organelle molecular basis for this species and enlarge the genetic resources of genus *Salvia*.

The *S. daiguii* individual was collected from Zhangjiajie, Hunan, China (GPS: 29°2′7.60′′N, 110°29′12.19′′E, voucher S0297, deposited at Herbarium of Shanghai Chenshan Botanical Garden (CSH)). DNA was extracted from its leaf using DNA Plantzol Reagent (Invitrogen, Carlsbad, CA). The raw data were generated using the Illumina platform (Illumina Inc., San Diego, CA). In total, about 3.8 G high-quality clean reads (150 bp PE read length) were obtained with adaptors trimmed. Following Liu et al. (2017, 2018), the complete chloroplast genome was assembled with *rbcL* gene as seed using software Novoplasty (Dierckxsens et al. 2017). Aligning and annotation were conducted by GeSeq (Tillich et al. 2017) and Geneious Prime (Biomatters Ltd, Auckland, New Zealand).

The complete chloroplast genome of *S. daiguii* (GenBank accession No. MT901216) has a length of 151,434 bp with a typical circle structure and a GC content of 38%. It consists of a large single-copy region (LSC, 82,753 bp, 36.1% GC content), a small single-copy region (SSC, 17,576 bp, 32.1% GC content), and two inverted repeat regions (IR, 25,553 bp, 43.1% GC content). In total, there were 134 genes in *S. daiguii*, including 88 protein-coding genes, eight *rRNA* genes, and 37 *tRNA* genes. Among them, six protein-coding genes (*rpl*2, *rpl*23, *ycf*2, *ycf*15, *ndh*B, and *rps*7), seven *tRNA* genes (*trn*I-CAU, *trn*L-CAA, *trn*V-GAC, *trn*I-GAU, *trn*A-UGC, *trn*R-ACG, and *trn*N-GUU) and all four *rRNA* genes (*rrn*16, *rrn*23, *rrn*4.5, and *rrn*5) have two copies. Nine of these protein-coding genes (*rps*16, *atp*F, *rpo*C1, *pet*B, *pet*D, *rpl*16, *rpl*2, *ndh*B, and *ndh*A) have one intron each and two (*ycf*3 and *clp*P) of them have two introns.

Seventeen species with available chloroplast genomes in Lamiaceae were selected to study the phylogenetic placement of *S. daiguii* (Figure 1). The sequence alignment was conducted by MAFFT version 1.3 (Katoh and Standley 2013). We drew phylogenetic tree by the software IQ-TREE (Nguyen et al. 2015) with 5000 bootstrap replicates and TVM + F+R2 model. The result of phylogenetics analysis showed that *S. daiguii* is closely related to *S. miltiorrhiza*.

**Figure 1.**
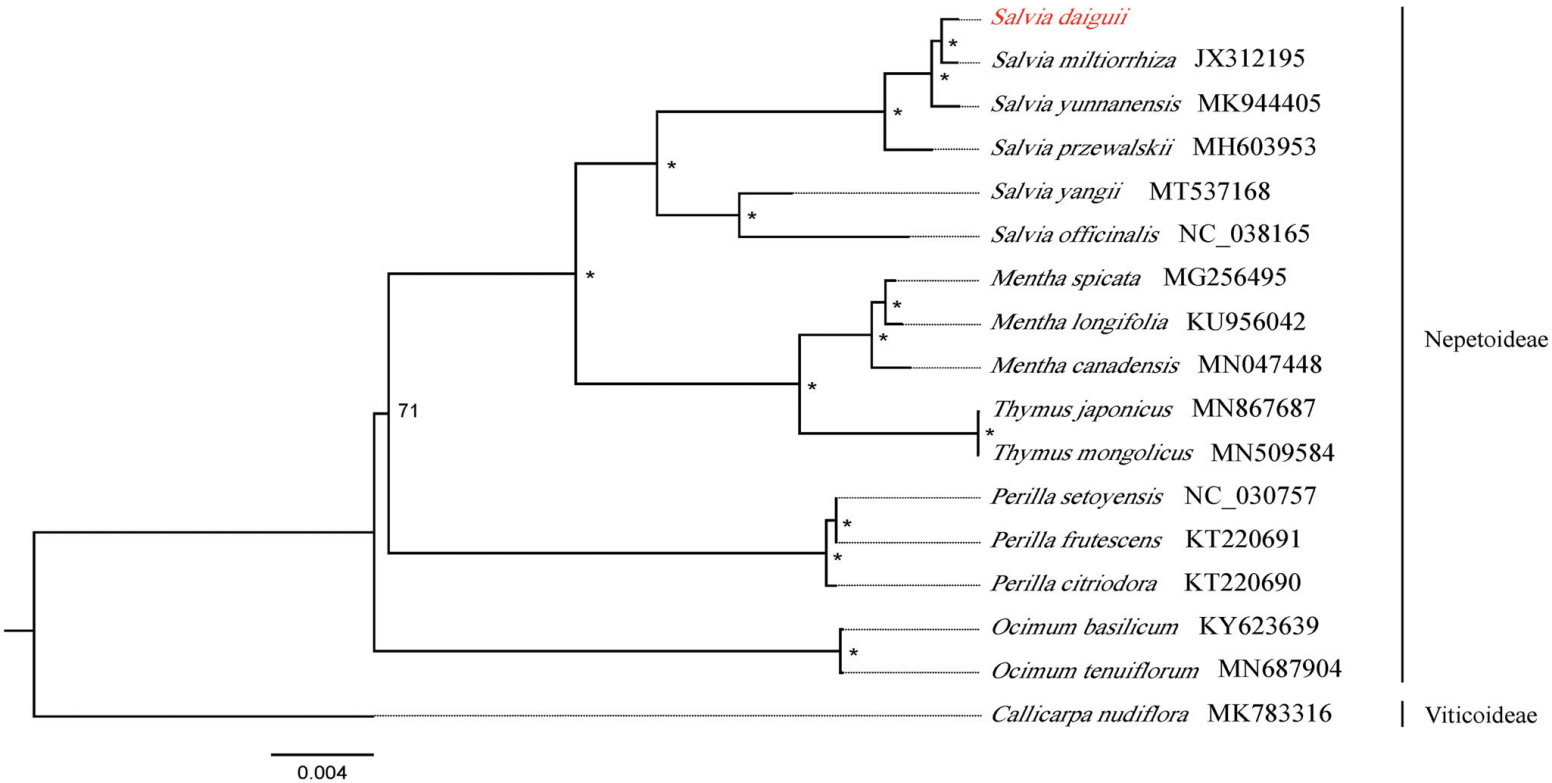
The phylogenetic tree based on 17 complete chloroplast genome sequences in Lamiaceae (accession numbers were listed behind each taxon. ‘*’ indicates the bootstrap support values are 100).

## Data Availability

Raw sequencing data have been deposited in SRA (SRR12667953). The DNA matrix and phylogenetic tree that support the findings of this study are openly available in GitHub at https://github.com/andresqi/Zhejiangjie-sage-chloroplast-genome.
